# Experiments with the Ligature on Animals

**Published:** 1852-03

**Authors:** J. Piernans

**Affiliations:** San Louis Potosi, Mexico


### ARTICLE II.

### *Experiments with the Ligature on Animals.*

Doctor Hester:

*Dear Sir:*—At your requests, I give you a succinct description of experiments made by me on living animals. The following is a faithful and correct account of said experiments, with their bearings on the actual state of Physiology and Pathology, etc.

Some years ago (I was then a student in the Charity Hospital of New Orleans) I noticed repeatedly, that patients dying in the very last stage of Phthisis Pulmonalis, offered at post mortem examination, strong thick cords crossing the cavernous hollows made by the progress of the disease. Upon close examination, I found that these cords were the pulmonary arteries obliterated in that part of the lungs. Such a pathological fact suggested the idea of applying this natural process of obliteration of the arteries in the cure of aneurism. During my stay in Mexico, I have been able to make experiments on living animals, and such experiments have confirmed the views I entertained on the subject.

On three living sheep, I took up one after the other the following arteries: The two carotids, and the two femoral.

After the first week, I noticed in all three an accelerated process of cicatrization, without any apparent suppuration, although the wounds had not been united by sutures, or any other means. At that time (8th day) I dissected in one the part where the ligature had been applied. Here I must say, that instead of using the ordinary silk ligatures employed in the operation of aneurism, and instead of tying the arteries (as is usually done in the operation of aneurism) tight enough to cut their inner coats, I used the common tape, and pushed it loosely around the artery, as is done in the case of a seton.

After a minute dissection, I noticed there was no perceptible pulsation at the distal side of the artery. I withdrew the ligature quite easily, as it did not press strongly on the artery. I could not perceive as yet any circulation in the above men.

tioned portion of the artery. I then cut the artery across, and observed that it was completely blocked up by a thick coagulated blood (the clot observed after tying an artery in the usual way.) Withdrawing the clot, a jet of genuine arterial blood came out.

The week after, (16th day) I dissected the neck and leg of the second sheep, and found that the wound was completely cicartrized. There was, as in the first case, no perceptible circulation in the artery below the seat of the ligature. Withdrawing the ligatures, there was no pulsation; cutting the artery, no blood came out; the clot was firmer, and adhered to the walls of the artery. I detached the clot with a little more difficulty than in the first sheep, and arterial blood came out.

In the third sheep, (on the 22d day) the clot was more strongly attached to the walls of the artery, and more firm, than in the two first instances.

In none of these three sheep could I notice any suppuration.

Thinking that some inflammation and suppuration would hasten the obliteration of the arteries, and render it more perfect, I performed successively the very same operatiou on two more sheep, three dogs and one calf. Instead of using simply the tape line, as I had done in the first cases, I applied to it some strong precipitate ointment, and took a great deal of care in bringing daily a fresh portion of the tape line in contact with the artery, and the parts surrounding it. It was with difficulty that I produced inflammation and a little suppuration in the sheep, but readily produced it in two dogs and in the calf.

After the 17th day, the obliteration of the arteries was perfect in all the sheep, the dogs, and in the calf.

Now, what is the bearing of these experiments in the operation for aneurism—especially tn the large arteries? Evidently, if performed on the human being, as I performed it on the living animals, there is not the slightest risk of secondary hæmorrhage; which, consequently, adds considerably to the chances of success, considering that in man inflammation and suppuration is more easily produced than in animals; such inflammation would, at the same time, be propagated to the different coats of the arteries, and, consequently, promote much quicker the obliteration of the arteries.

Yours, respectfully, J. Piernas, M. D.

*San Louis Potosi, Mexico*. 1851.

[*New Orleans Med. and Surg. Journal.*

